# Sleep Apnea and Hypoventilation in Patients with Down Syndrome: Analysis of 144 Polysomnogram Studies

**DOI:** 10.3390/children4070055

**Published:** 2017-06-30

**Authors:** Zheng Fan, Mihye Ahn, Heidi L. Roth, Leping Li, Bradley V. Vaughn

**Affiliations:** 1 Department of Neurology, University of North Carolina at Chapel Hill, Chapel Hill, NC 27599, USA; Hroth@neurology.unc.edu (H.L.R.); vaughnb@ad.unc.edu (B.V.V.); 2 Department of Mathematics and Statistics, University of Nevada, Reno, NV 89503, USA; mihye98@gmail.com; 3 Biostatistics and Computational Biology, National Institute of Environmental Sciences, Research Triangle Park, NC 27709, USA; li3@niehs.nih.gov

**Keywords:** Down syndrome, hypoventilation, sleep apnea and sleep disordered breathing

## Abstract

Patients with Down syndrome (DS) are at risk for both obstructive sleep apnea (OSA) and central sleep apnea (CSA); however, it is unclear how these components evolve as patients age and whether patients are also at risk for hypoventilation. A retrospective review of 144 diagnostic polysomnograms (PSG) in a tertiary care facility over 10 years was conducted. Descriptive data and exploratory correlation analyses were performed. Sleep disordered breathing was common (seen in 78% of patients) with an average apnea-hypopnea index (AHI) = 10. The relative amount of obstructive apnea was positively correlated with age and body mass index (BMI). The relative amount of central sleep apnea was associated with younger age in the very youngest group (0–3 years). Hypoventilation was common occurring in more than 22% of patients and there was a positive correlation between the maximum CO_2_ and BMI. Sleep disordered breathing, including hypoventilation, was common in patients with DS. The obstructive component increased significantly with age and BMI, while the central component occurred most in the very young age group. Due to the high risk of hypoventilation, which has not been previously highlighted, it may be helpful to consider therapies to target both apnea and hypoventilation in this population.

## 1. Introduction

Down syndrome (DS) is the most common genetic disorder [1] in humans and sleep disordered breathing is common among this population. A prior study estimated the obstructive sleep apnea (OSA) prevalence in this population to be 66% [2,3,4,5]. Patients with DS have multiple clinical characteristics that predispose them to OSA including midface hypoplasia, narrow nasopharynx, small pharynx, relative macroglossia and retroglossia, and increased body mass [6,7]. They also have features that can predispose them to central sleep apnea including hypotonia, central nervous system (CNS) impairment, and acid reflux. Additionally, these patients are at risk for hypoventilation, a risk which can be promoted by clinical features of DS including congenital heart disease, smaller lung volumes, hypotonia, and changes in respiratory control in the setting of elevated body mass index (BMI) [6,7]. Little is known about how sleep disordered breathing changes in DS as patients grow older and how changes in clinical features of the DS phenotype might impact sleep disordered breathing over time. Parameters that change as patients grow (e.g., maturity of the CNS, airway structure, weight, and muscle tone) and how they might result in changes in risk of sleep disordered breathing over time [8]. The risk for hypoventilation in DS and any changes in hypoventilation over time have also not previously been well characterized in this population and could have some important implications for treatment choices.

We aimed to investigate how both obstructive apnea and central apnea change over time in patients with DS as well as to evaluate the risk for hypoventilation in these patients. We hypothesized that the risk for obstructive apnea would increase with age due to relative increase in BMI, but the risk for central apnea would decrease due to improved maturity of the respiratory control systems. We also hypothesized that patients would be at increased risk for hypoventilation at all ages due to cardiopulmonary conditions, residual inefficiency of respiratory control systems in the setting of elevated BMI, and hypotonia. Finally, we planned to explore correlations between sleep apnea, hypoventilation, and other indices from the polysomnograms (PSG) data to investigate how sleep related parameters might be different for patients with differing clinical characteristics and respiratory findings.

## 2. Results

### 2.1. Demographics and Descriptive Data 

A total of 168 diagnostic studies were identified that were performed in patients with DS over a 10-year span. Among these, 164 were diagnostic PSGs and 4 were split-night studies from which the diagnostic portion of the study was included. Twenty-four studies were excluded for the following reasons: a total sleep time less than 240 min or a technically inadequate study (e.g., lack of reliable breathing sensors). Demographics and basic polysomnographic characteristics of the 144 PSGs are summarized in Table 1. Among these 144 studies, 14 studies were done in DS patients of more than 18 years (20–45 years) of age. These older patients with DS had severe sleep disordered breathing with an average apnea-hypopnea index (AHI) of 46.

The study included patients with ages ranging from 0.25 year (3 months) to 45 years. The mean and median ages were 7.6 and 4.7 years respectively. Overall, the descriptive statistics indicated our sample was skewed toward the younger age group. Sleep disordered breathing was common in the group as a whole (78%), and the average AHI was 9.8. We also found that the average time spent with oxygen saturation < 88% was 14.3 min. The average maximum CO_2_ measurements was 49.8 torr with a positive correlation between time spent with O_2_ saturation < 88% and maximum CO_2_ (*p*-value = 0.09). These data suggested that patients with DS are not only at risk for hypoxemia but also for hypoventilation syndrome and/or hypoxemia. 

### 2.2. Obstructive Sleep Apnea

Obstructive sleep disordered breathing was common in this cohort. A total of 78% of patients met the criteria for OSA diagnosis (mild OSA: 34% (49/144); moderate OSA: 21% (30/144); severe OSA 24% (34/144); absence of OSA 22% (31/144)). Almost half of the group (45%) had moderate to severe OSA. 

**Figure 1:**
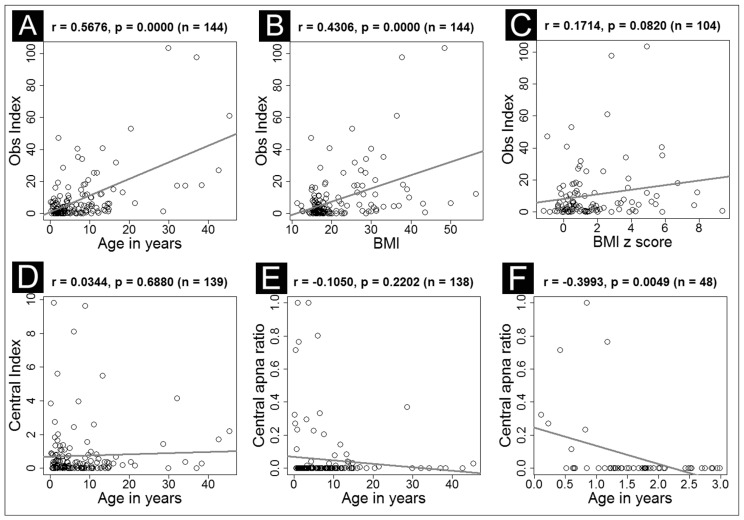
Correlation between obstructive apnea index and age (**A**) and BMI (**B**) and BMI *z* score (**C**). Correlation of central apnea index and age in all subjects (**D**); central apnea ratio and age in all subjects (**E**) and central apnea ratio and age in children ≤ 3 years (**F**). Pearson correlation coefficient (*r*), its associated *p*-value (*p*), and the number of subjects (*n*) are listed in the header of each plot.

The exploratory correlation analysis revealed that overall sleep disordered breathing (using AHI as a marker) was positively correlated with the following characteristics: older age, higher BMI, more severe oxygen desaturation during the night, and longer time spent O_2_ < 88%. These associations remained statistically significant after adjustment for age, except for BMI (Table 2). The obstructive index was positively correlated with age and BMI and trending with BMI *z* score (Figure 1A–C). 

### 2.3. Evolution of Obstructive versus Central Component

We examined the obstructive component and central component of apnea and their relationships to age. It was clear that the obstructive component (both obstructive index and obstructive ratio) had a strong age effect in that high obstruction was associated with increased age. (Figure 1A). However, the central component did not show any age effect when applied to all age groups (Table 3). On examination of the raw data, it appeared that central apnea evolved differently across age groups. We performed a further analysis on the age subgroups and found that central apnea was only correlated with age in the very young age group (0–3 years) with the central apnea ratio of the younger age group being higher (Figure 1F). We did not see such a correlation in the older age group (Figure 1D,E). 

### 2.4. Hypoventilation Risk

There were 32 patients with clear evidence of hypoventilation. Fifty studies had insufficient data for determination of hypercapnia. This led to a conservative estimation of the rate of hypoventilation for all studies (assuming all missing studies did not have hypoventilation) of 22% (32/144) (Table 4). Further analysis showed that although the hypercapnia indices (baseline CO_2_, mean CO_2_, and max CO_2_) were not correlated with age or BMI *z* score, BMI was marginally correlated with the maximum CO_2_ (*p* = 0.051, Table 5). Furthermore, maximum CO_2_ measurements were negatively correlated with the lowest oxygen desaturations, and there was a trend for a positive correlation (*p* = 0.09) with time spent with O_2_ < 88% and the need for supplemental oxygen and baseline respiratory rate (Table 5). No statistically significant correlations were found between obstructive index, central index and CO_2_ measurements. 

### 2.5. Sleep Structure

In this cohort, several indices of sleep structure showed clear relationships to age. Total sleep time, sleep efficiency, stage N3%, and stage R% all decreased with age, whereas rapid eye movement (REM) latency increased with age. Most of these sleep parameters were not associated with oxygen or CO_2_ measures except stage N3%, which was correlated with higher average CO_2_ measurement (data not shown). 

## 3. Discussion

Our study is one of the largest polysomnogram studies in patients with DS and included 144 studies over a span of 10 years. Our analysis found a high rate of sleep disordered breathing in these patients and a degree of apnea that is often severe [2,3,4,5,9,10,11,12]. Seventy-eight percent of patients with DS in our study had OSA, slightly higher than the 66% rate reported by Maris and colleagues [13]. We decided to use the Centers for Disease Control and Prevention (CDC) BMI chart to adjust BMI as Hatch–Stein et al. concluded that the CDC BMI growth chart percentiles are a better indicator of excessive adiposity than the new DS-specific BMI charts for patients with DS [14,15]. We used CDC age and gender adjusted BMI *z*-scores and the raw BMI values in the analysis. Overall, AHI and obstructive apnea index were positively correlated with both age and BMI; however, after adjustment for age, BMI was no longer correlated with AHI. These findings suggest a potential interaction between age and BMI in that age might contribute significantly to BMI. 

It is not surprising that sleep disordered breathing is strongly correlated with age in patients with DS. This correlation was largely due to the increased obstructive component of sleep disordered breathing. Although the central apnea index did not significantly correlate with age, the central apnea ratio was increased in the younger end of the youngest subgroup (0–3 years), mainly in infancy and early toddlerhood. These findings support our hypothesis that, in the very young DS age group, there is an increased propensity for central apnea which could be due to increased incidence of hypotonia and immature respiratory control in this DS age group. 

Our study is among the first to demonstrate that hypoventilation is common in patients with DS. At least 22% of patients had hypoventilation in this study. The cause for this is unclear. Hypercapnia was not correlated with age or AHI, but maximum CO_2_ measurements were negatively correlated with the lowest O_2_ desaturation and had trend for a correlation with BMI, time spent with O_2_ < 88% and the need for supplemental O_2_. (Table 5). Hypoventilation and sleep apnea (both obstructive and central apneas) can and often overlap in general populations, but it is worth focusing separate attention on the hypoventilation component as it may require different treatment and may explain why traditional surgical treatment may not be successful in this population. Our analysis suggested that there was a trend between the maximum CO_2_ correlation and BMI. We speculate that obesity might be a contributing factor to the hypoventilation found in these patients. The high risk of hypoventilation in this patient population has not been extensively discussed in the literature, despite being reported by Marcus et al in 1991 [16]. Yet, this risk may have practical implications that could affect choice of treatment options. Awareness of hypoventilation could result in earlier consideration of positive airway pressure (PAP) therapy or possibly earlier bi-level PAP therapy that could benefit this population. Further studies are needed to evaluate whether earlier use of PAP therapy would improve clinical outcomes in this population of patients. 

Moreover, it is also worth noting that patients with DS have significant sleep related hypoxemia. We found that subjects spent an average time of 14.3 min with oxygen saturation < 88%. It is known that different populations have different capacities for maintaining oxygen saturations in response to apnea pauses [17]. Patients with DS have been reported to have prolonged postoperative desaturations. In a mouse model of DS (Ts65Dn), an increased incidence of intermittent hypoxemia was observed [18]. We speculate that certain features in patients with DS may contribute to the susceptibility for hypoxemia such as low functional reserve capacity (FRC) for gas exchange related to obesity, underlying hypotonia, and the comorbidity of cardiac disease which may reduce vascular flow rates. The underlying mechanism for this hypoxia is not clear. Our analysis suggests that the overall AHI may underestimate the severity of the sleep disordered breathing in patients with DS. 

Limitations of this study include the retrospective nature of data collection, and the acquisition from a tertiary health care center which may skew data toward the more severe spectrum. 

## 4. Materials and Methods

### 4.1. Subjects and Study Protocol

Ethical approval for this retrospective data review was granted by the University of North Carolina at Chapel Hill Institutional Review Board (IRB14-2722). A retrospective “in laboratory” PSG data review was conducted for patients with diagnosis of “Down syndrome” or “Trisomy 21” between January 2005 and December 2014 in an American Academy of Sleep Medicine (AASM) accredited sleep laboratory in a tertiary care hospital. 

All subjects with the diagnosis of DS who underwent overnight diagnostic PSG (diagnostic or split-night) were included. The diagnostic portion of the split-night was analyzed together with the diagnostic studies. Only PSGs with incomplete data were excluded. Overnight PSG included at least six channel electroencephalograms, two electrooculograms, submental and bilateral tibialis surface electromyograms, an electrocardiogram, recordings from thoracic and abdominal excursion belts, oronasal airflow measures (thermocouples and nasal pressure), finger oximetry, and end tidal CO_2_ or transcutaneous CO_2_ measurements for the majority of patients. The studies were manually scored and interpreted by physicians who were board certified by the American Board of Sleep Medicine using the AASM manual for studies after September 2007 and the guidelines from the Sleep Disorders Atlas Task Force of the American Sleep Disorders Association for studies prior to 2007 [19,20]. For both time frames, the respiratory events were required to be a minimum of two breaths in duration. Hypopneas were required to be associated with a 3% desaturation or arousal. Apnea severity was defined as follows: for patients who were younger than 18: mild OSA: AHI ≥ 1 and < 5; moderate OSA: AHI ≥ 5 and < 10; and severe OSA: AHI > 10. For patients who were 18 and older, mild OSA: AHI ≥ 5 and <15; moderated OSA: AHI ≥ 15 and <30; and severe OSA: AHI > 30 [21]. For patients who were younger than 18: hypoventilation is defined as > 25% time in sleep spent with partial pressure (tension) of carbon dioxide (pCO_2_)or surrogate measurements > 50 mmHg and for patients who were 18 and older, hypoventilation was defined as pCO_2_ or surrogate measurements > 55 mmHg for ≥ 10 min or > 50 mmHg and with a more than 10 mmHg increase in sleep in comparison to awake [21]. 

### 4.2. Data Analysis 

Descriptive data, including means and standard deviations, were calculated from the demographic and sleep characteristics of the population cohort. We computed Pearson correlation coefficients to examine the relationships between PSG indices and other parameters. Variables included in the analysis were: age, BMI, BMI *z* score [22], oxygen supplement, heart rate, respiration rate, baseline CO_2_, baseline oxygen saturation, total sleep time (TST), sleep efficiency, sleep onset latency (SOL), REM onset latency (REMOL), N3%, REM%, apnea-hypopnea index (AHI), obstructive apnea index, obstructive ratio (obstructive index/AHI), central apnea index, central apnea ratio (central apnea index/AHI), hypopnea event number, respiratory disturbance index (RDI), lowest oxygen desaturation, average oxygen saturation, time spent <88%, mean CO_2_, maximum CO_2_, periodic limb movement index, arousal index. Multiple comparisons were corrected. To evaluate the longitudinal characteristics of these features, we adopted a linear mixed model. We used R 3.1.2 software to fit models and perform all analyses [23]. 

## 5. Conclusions

In summary, our study showed that patients with DS were at risk for OSA, hypoventilation, and hypoxemia, and there was an increased propensity for central apnea in the very young patients (<3 years). Treatments that improve ventilation such as PAP therapy may need to be considered earlier for management in patients with DS, especially when improvement is suboptimal after traditional interventions such as surgery. 

## Figures and Tables

**Table 1:** Demographics data and basic polysomnographic characteristics (*n* = 144).

	Mean	SD	*n* *	Min	Max
Gender	0.6	n/a	144	n/a	n/a
Age in years (median)	7.6 (4.7)	8.5	144	0.1	45.4
BMI	21.3	8.1	144	11.3	56.4
BMI *z* score	1.7	2.1	104	−1.2	9.4
Heart rate	99.6	19.4	124	56.0	140.0
Respiratory rate	24.7	10.8	137	12.0	87.0
CO_2_ (torr)	42.2	6.5	105	24.0	63.0
O_2_ %	96.0	2.8	122	77.0	99.0
TST (min)	425.5	71.9	144	242.0	643.5
S Efficiency (%)	84.6	9.8	144	50.1	99.0
SOL	20.0	25.0	144	0.0	144.0
REM latency (min)	159.0	94.6	117	7.5	449.0
N3 (%)	29.6	12.3	144	0.2	68.4
REM (%)	15.0	9.1	144	0.0	50.3
AHI	9.8	16.0	144	0.0	103.4
Obstructive index	8.6	15.5	144	0.0	103.4
Obstructive ratio	0.7	0.3	144	0.0	1.0
Central Index	0.7	1.6	139	0.0	9.8
Central apnea ratio	0.1	0.2	138	0.0	1.0
RDI	9.7	17.5	39	0.0	103.5
Average O_2_ Sat (%)	95.2	2.2	143	82.9	99.0
Lowest Desats (%)	82.7	9.2	138	51.0	96.0
Time < 88% (min)	14.3	28.5	142	0.0	192.9
Mean CO_2_	43.6	9.0	20	34.0	75.5
Max CO_2_	49.8	5.5	96	36.0	60.0
PLMI	2.3	6.7	144	0.0	58.0
Arousal index	8.7	5.6	137	0.0	38.6

AHI: apnea/hypopnea index; BMI: body mass index; PLMI: periodic limb movement index; RDI: respiratory disturbance index; REM: rapid eye movement; REMOL: rapid eye movement sleep onset latency; SD: standard deviation; SOL: sleep onset latency; TST: total sleep time. * Total sample size was 144 and those fewer than 144 were resulted from missing data for the specific parameters.

**Table 2:** Correlation between sleep disordered breathing indices and other characteristics.

		Age	BMI	BMI *z* Score	Lowest O_2_ Desats	Time O_2_ < 88%
AHI (age unadjusted)	*r*	0.57	0.41	0.14	−0.40	0.30
	*p*	<0.001 *	<0.001 *	0.15	<0.001 *	<0.001 *
AHI (age adjusted)	*r*	-	0.12	0.12	−0.35	0.23
	*p*	-	0.16	0.24	<0.001 *	0.01 *

* Denotes *p*-value ≤ 0.05. AHI, apnea/hypopnea index and BMI, body mass index.

**Table 3:** Correlation between obstructive/central apnea parameters and age.

		All Ages	Age Break Down of Pediatric Patients		
			Age (0–3 Years)	Age (4–8 Years)	Age (9–16 Years)
Obstructive Index	*r*	0.57	0.01	0.16	−0.10
	*p*	<0.001 *	0.97	0.29	0.53
Obstructive ratio	*r*	0.29	−0.09	−0.10	0.13
	*p*	<0.001 *	0.53	0.52	0.43
Central index	*r*	0.03	−0.19	0.14	−0.09
	*p*	0.69	0.19	0.38	0.60
Central ratio	*r*	−0.11	−0.40	−0.03	0.16
	*p*	0.22	<0.001 *	0.86	0.35

* Denotes *p*-value ≤ 0.05.

**Table 4:** Hypoventilation frequency.

Hypercapnia (%)	Normal CO_2_	No Sufficient CO_2_ Data	Total Studies
32 (22%)	62	50	144

Hypoventilation is defined as ≥ 25% of the total sleep time spent with end tidal CO_2_ (ET CO_2_) or transcutaneous CO_2_ (tcCO_2_) measurements at level above 50 torr.

**Table 5:** Correlation between Ventilation Parameters and CO_2_.

		Age	BMI	BMI *z* Score	AHI	O_2_	Avg. O_2_	Lowest O_2_ Desats	O_2_ < 88%	Suppl. O_2_	Resp. Rate
**Baseline CO_2_**	*r*	−0.09	−0.001	−0.04	0.03	0.01	0.06	−0.08	0.05	0.25	0.11
	*p*	0.35	0.99	0.70	0.79	0.92	0.54	0.41	0.63	0.01 *	0.29
**Mean CO_2_**	*r*	−0.22	0.11	−0.18	−0.02	0.16	0.20	−0.36	−0.03	0.31	0.56
	*p*	0.36	0.63	0.44	0.94	0.52	0.40	0.13	0.88	0.21	0.01 *
**Max CO_2_**	*r*	0.05	0.19	0.17	0.13	0.01	−0.09	−0.24	0.17	0.10	−0.01
	*p*	0.59	0.051 **	0.10	0.24	0.94	0.37	0.01 *	0.09 **	0.08 **	0.95

* Denotes *p*-value ≤ 0.05, ** denotes *p*-value < 0.10 and > 0.05.
